# Identification of low health and cancer literacy in oncology patients: a cross-sectional survey

**DOI:** 10.1007/s00520-021-06164-2

**Published:** 2021-05-03

**Authors:** Amelia Hyatt, Allison Drosdowsky, Trista Koproski, Donna Milne, Meri Rametta, Geri McDonald, Tanya McKenzie, Sarah-May Blaschke

**Affiliations:** Peter MacCallum Cancer Centre, 305 Grattan Street Parkville, Melbourne, VIC Australia

**Keywords:** Cancer Health Literacy, Patient Participation, Healthcare Communication, Health Systems

## Abstract

**Objective:**

Health literacy is a significant public health concern, particularly given the increased complexity of chronic disease health management and health system navigation, and documented associations between low health literacy and poor health outcomes. This study therefore aimed to identify the proportion and characteristics of outpatients visiting a specialist cancer hospital who report low health literacy and/or low cancer health literacy.

**Method:**

This study used a cross-sectional survey administered verbally with patients attending a specialist cancer hospital located in Melbourne, Australia over a two-week period. Process data on conducting health literacy screening within a clinical setting was collected.

**Results:**

Those identified with inadequate general health literacy were different to those identified with low cancer-specific health literacy, although overall both proportions were low. Cross-sectional screening of patients was difficult, despite utilising verbal surveying methods designed to increase capacity for participation.

**Conclusion:**

Health literacy screening using the tools selected was not useful for identifying or describing patients with low health literacy in this setting, given the disparity in those categorised by each measure.

**Practice Implications:**

Until the theoretical construct of health literacy is better defined, measurement of health literacy may not be clinically useful.

**Supplementary Information:**

The online version contains supplementary material available at 10.1007/s00520-021-06164-2.

## INTRODUCTION

Older, seminal definitions of health literacy describe the universal and necessary abilities required by patients to successfully engage with the healthcare system, with a strong emphasis on underlying literacy and numeracy skills [1–6]. Those who struggle with the demands of managing their health are classified as having low or inadequate health literacy [6, 7]. Research has consistently identified poorer health outcomes for individuals with low health literacy [7–9], and this is now recognised as a significant public health problem [2]. Understanding the health literacy of the population in order to improve health service delivery is a policy priority for both state and federal governments in Australia [10].

As diagnostic and treatment options for chronic diseases such as cancer improve, patients are increasingly required to navigate complex healthcare systems, and manage treatment regimens at home [11–13], health literacy has subsequently been broadened to encompass knowledge regarding disease-specific health management and care pathway navigation [14, 15]. More recently, definitions of health literacy have been further expanded to include environmental, social, and system factors, although the focus has predominantly remained on individual ability to access, understand and use health related information [16]. Identifying the level of health literacy and/or cancer health literacy among patients is important to ensure that healthcare systems provide adequate cancer care [14, 17]. However, in considering newer conceptual models of health literacy and related system level programs aiming to reduce low health literacy, accurate understanding of population characteristics and needs are required for these to be appropriate and effective [16].

There are a large number of instruments ostensibly designed to identify low health literacy; utilising either subjective measures such as self-report of skills and capacity, or performance-based objective measures which aim to test participants’ knowledge, literacy or numeracy in a simulated health context or setting [18]. Screening tests such as the self-report Brief Health Literacy Screening Tool (BRIEF) and the 6-Item performance-based Cancer Health Literacy Screening Tool (CHLT-6) have both been designed to identify inadequate health literacy in a clinical setting [1, 15, 19, 20].

Comparing the performance of subjective and objective measures in identifying patients with low health literacy using a cross sectional survey approach is important to determine which may be useful and practicable in an applied tertiary healthcare setting. Likewise, as literacy and numeracy skills underpin many health literacy definitions and measurement tools [16], understanding whether self-report of these skills can be used as a proxy for health literacy in busy clinical settings is also of interest. This study therefore aimed to identify the proportion and characteristics of outpatients visiting a specialist cancer hospital who report low health literacy and/or low cancer health literacy using the BRIEF and the CHLT-6. Associations between health literacy and participant characteristics including self-reported confidence in reading, writing and mathematics were assessed. Process data, including patient feedback on conducting health literacy screening using a cross-sectional approach within a clinical setting are reported.

## METHODS

### Participants and setting

This study surveyed a convenience sample of patients who attended a specialist cancer hospital located in Melbourne, Australia over a two-week period. Inclusion criteria comprised: adult patients attending outpatient clinics and the day therapy unit, who could speak and understand English. Patients who did not have a diagnosis of cancer were excluded. This study was approved by the Peter MacCallum Human Research Ethics Committee (approval no: LNR/18/PMCC/72).

### Procedures

In preparation for recruitment, members of the research team conducted 30-minute data collection training sessions with twenty-five volunteers from the cancer hospital. The trained volunteers then approached patients in the clinic waiting rooms or while they were receiving treatment in the day therapy unit. Following consent, volunteers administered measures using a verbal, face-to-face interview format to facilitate participation of those with low or no reading and/or writing ability. A laminated item response sheet was available for participants who wished to read along while questions and response options were read aloud. Responses were recorded using a study-specific hard-copy case report form. Volunteers recorded the date and noted counts for those who declined and those who had been previously approached. Hard-copy data were stored securely in the research department and entered into a REDCap database by a trained data manager [21].

### Measures

The survey consisted of five sections:

#### Demographic survey

A short study-specific survey was designed to collect sociodemographic information and clinical characteristics, including: age, gender, educational level, occupation (or former occupation), postcode, cancer diagnosis, and time since diagnosis.

#### Six-Item Cancer Health Literacy Test (CHLT-6)

The CHLT-6 is a psychometrically validated measure designed to screen and identify individuals with low cancer health literacy [15]. It comprises six questions, and categorises individuals as either adequately or inadequately cancer health literate [15].

#### Brief Health Literacy Screening Tool (BRIEF)

The BRIEF is a psychometrically validated health literacy screening tool [17]. It was developed using qualitative data from patients with limited health literacy who identified 5 key domains of concern: completing medical forms, navigating the healthcare system, interacting with providers, following medication instructions and reading appointment slips [1]. The BRIEF categorises individuals as having inadequate, marginal or adequate health literacy.

#### Literacy and numeracy self-report

Participants were asked to describe their self-evaluated levels of confidence in reading, writing and in maths as free text responses recorded by the volunteers.

#### Field notes

Three items were completed by the volunteers. The first asked whether the patient used the item response sheet as a reference, and the second asked whether the patient had assistance with survey completion. The final question was open-ended and asked volunteers to note any additional information regarding the survey delivery or completion process, or feedback provided by patients regarding the project or health literacy/health information provision.

### Quantitative data analysis

A coding framework was developed to categorise each of the following variables: diagnosis, time since diagnosis, education, occupation, and self-reported confidence in reading, writing and mathematics. Discrepancies were discussed by AH, SB, TK, and AD until consensus was reached. Verbal responses for self-reported confidence in reading, writing and mathematics were dichotomised into ‘no reservations’ or ‘some/significant reservations’.

The patient-reported health literacy measures were scored according to their protocols [15, 17, 20]. Only respondents with complete data for the CHLT-6 were given a health literacy score, where responding correctly to five or more of the six items reflects ‘adequate’ health literacy. In the absence of instructions for how to manage missing data for the BRIEF, respondents were given a health literacy score if they completed more than fifty percent of the four items, or three or four items. Scores for participants with incomplete responses were calculated based on the average score for the completed items.

Survey data were reported descriptively, using means/standard deviations, medians/interquartile ranges or counts/frequencies as appropriate. Relationships between health literacy measures and demographic variables were examined with the reporting of the appropriate effect size, specifically the phi coefficient for two by two tables, Cramer’s V for larger tables, Cohen’s d for comparing two means and eta squared for comparison of more than two means. Agreement between the two methods of health literacy screening was assessed using Cohen’s kappa. This method requires the same number of outcomes for each measure. Therefore, participants who scored ‘marginal’ on the BRIEF were classified into ‘adequate’ and ‘inadequate’ separately and two analyses were performed. All analyses were performed in R [22].

### Qualitative data analysis

Qualitative data generated from free text items were analysed using qualitative content analysis methodology, as it is useful for examining open-ended patient experience data generated with the intent of informing clinical practice [23]. Data were exported into Microsoft Excel for analysis by AH, with identifying information removed [24]. All comments were reviewed and categorised by context (patient/volunteer) and/or intent (field note by volunteer or comment from patient). Themes were generated. All codes were reviewed by SB and inconsistencies discussed and resolved.

## RESULTS

### Sample

During a two-week period, 3164 individuals were recorded as attending the outpatient specialist clinics, of these, 457 individuals were approached (14%). Of those approached, 92 declined (20%) and 365 agreed to take part (80%). The final sample was 345 after removing respondents who only completed the demographic portion of the survey (n=6), participants with a non-cancer diagnosis (n=10), and surveys that were not completed by the patient themselves but by a family member or friend on their behalf (n=4). Most participants used the provided item response sheets as a reference (n=244, 71%) and did not receive assistance (n=229, 66%).

Volunteers estimated completing the verbal survey took approximately 20 minutes per patient. Clinics ran for approximately three hours; therefore volunteers were able to each complete an average of nine patient surveys per clinic. At each clinic, a minimum of one to a maximum of five volunteers were available to conduct surveys.

The demographic and clinical characteristics of respondents are shown in Table 1. The average age of participants was 61 years (standard deviation 14 years) and there were slightly more women than men (53% versus 47%). The sample comprised patients diagnosed with a variety of cancers, the most common being haematological (n=64, 19%), skin and melanoma (n=56, 16%) and breast (n=37, 11%).

**Table 1:** Demographic and clinical characteristics of participants (n=345)

**Age**		
Mean (SD)	60.5 (14.0)	
Median (IQR)	62 (52, 71)	
Range	18, 87	
**Gender**	**n**	**%**
Male	162	47.0
Female	183	53.0
**First language**		
English	301	87.2
Not English	38	11.0
Missing	6	1.7
Residence*		
Major City	232	67.2
Inner regional	87	25.2
Outer regional	21	6.1
Remote	0	0.0
International	1	0.3
Missing	4	1.2
**Highest level of education**		
None	1	0.3
Some primary school	1	0.3
Completed primary school	10	2.9
Some high school	100	29.0
Completed high school	67	19.4
Trade/TAFE college	36	10.4
Tertiary	111	32.2
Missing	19	5.5
**Cancer type**		
Haematology	64	18.6
Skin & Melanoma	56	16.2
Breast	37	10.7
Lung	26	7.5
Urology	22	6.4
Head & Neck	19	5.5
Gynaecological	18	5.2
Lower GI	16	4.6
Upper GI	12	3.5
Bone & soft tissue	11	3.2
Neurological	2	0.6
Cancer, other	38	11.0
Missing	17	4.9
No cancer diagnosis yet	7	2.0
**Time since diagnosis**		
≤ 6 months	87	25.2
> 6 months ≤ 5 years	178	51.6
> 5 years ≤ 10 years	32	9.3
>10 years	31	9.0
Missing	17	4.9

* The Accessibility/Remoteness Index of Australia (ARIA) was used to categorise patient-reported postcode data

### Brief Health Literacy Screening Tool (BRIEF)

Respondents’ health literacy as judged by the BRIEF is shown in Table 2. Six respondents (2%) did not complete enough items to be included. Of the 339 remaining, the majority showed ‘adequate’ health literacy (n=229, 68%), followed by ‘marginal’ (n=67, 20%) and ‘inadequate’ (n=43, 13%). Responses to each individual item are reported in Supplementary Appendix 1.

**Table 2:** Health Literacy Assessments (n=345)

**BRIEF Health literacy**	**n**	**%**
Inadequate	43	12.7
Marginal	67	19.8
Adequate	229	67.6
Missing	6	
**CHLT-6 Cancer health literacy**		
Inadequate	66	20.8
Adequate	252	79.2
Missing	27	
**Self-reported reading confidence**		
No reservations	283	89.0
Some or significant reservations	35	11.0
Missing	27	
**Self-reported writing confidence**		
No reservations	267	84.5
Some or significant reservations	49	15.5
Missing	29	
**Self-reported mathematics confidence**		
No reservations	230	72.8
Some or significant reservations	86	27.2
Missing	29	

### Six-Item Cancer Health Literacy Test (CHLT-6)

The scores of the CHLT-6 are shown in Table 2. Ninety-two percent of respondents (n=318) answered every item of the CHLT-6 and were therefore assigned a level of health literacy. The majority of these had ‘adequate’ health literacy (n=252, 79%). The responses to each individual CHLT-6 item are reported in Supplementary Appendix 2. Most responses to individual items were correct, with the proportion of correct responses ranging from 79% (item 5) to 96% (item 6).

### Self-reported literacy assessment

Participants were asked to self-assess their level of confidence with reading, writing and mathematics; these responses are shown in Table 2. Most respondents reported no reservations with their reading and writing abilities, however, 27% of the sample (n=86) reported some or significant reservations in relation to mathematics.

### Health literacy and cancer health literacy comparisons

Table 3 shows the relationship between the CHLT-6 and the BRIEF health literacy tools. The two tools do not appear to provide similar assessments of health literacy within this sample. Notably, over half of the respondents who had ‘inadequate’ health literacy using the CHLT-6 had ‘adequate’ health literacy on the BRIEF (n=37, 58%). Similarly, the level of agreement was slight (kappa=0.13) when the BRIEF ‘marginal’ category was combined with ‘inadequate’, and fair (kappa=0.24) when the ‘marginal’ category was combined with ‘adequate’.

**Table 3:** Relationships between health literacy measures and participant characteristics

	**BRIEF**				**CHLT-6**		
	Inadequate	Marginal	Adequate	Effect size*	Inadequate	Adequate	Effect size*
**Age**							
Mean (SD)	63.4 (14.4)	60.9 (14.8)	59.6 (13.7)	trivial	66.2 (12.9)	58.3 (13.6)	medium
Range	18, 87	19, 87	29, 87		29, 87	19, 87	
**Gender**	n (%)**	**n (%)**	**n (%)**		**n (%)**	**n (%)**	
Female	26 (14.4)	28 (15.6)	126 (70.0)	small	40 (25.8)	115 (74.2)	small
Male	17 (10.7)	39 (24.5)	103 (64.8)		26 (16.0)	137 (84.0)	
**Education**							
Did not complete high school	23 (21.3)	19 (17.6)	66 (61.1)	small	25 (26.6)	69 (73.4)	small
Completed high school	17 (8.0)	44 (20.7)	152 (71.4)		33 (15.9)	174 (84.1)	
**Language**							
English	29 (9.8)	56 (18.9)	212 (71.4)	medium	50 (17.7)	233 (82.3)	small
Other	13 (35.1)	10 (27.0)	14 (37.8)		13 (44.8)	16 (55.2)	
**Self-reported confidence**							
**Reading**							
No reservations	21 (7.5)	53 (18.9)	207 (73.7)	large	50 (17.8)	231 (82.2)	small
Reservations	16 (47.1)	7 (20.6)	11 (32.4)		15 (48.4)	16 (51.6)	
**Writing**							
No reservations	17 (6.4)	49 (18.5)	199 (75.1)	large	49 (18.5)	216 (81.5)	small
Reservations	20 (41.7)	11 (22.9)	17 (35.4)		16 (35.6)	29 (64.4)	
**Mathematics**							
No reservations	18 (7.9)	43 (18.9)	167 (73.2)	small	40 (17.5)	189 (82.5)	small
Reservations	19 (22.4)	17 (20.0)	49 (57.6)		25 (30.9)	56 (69.1)	
**Health Literacy measures**							
**CHLT-6**							
Adequate	17 (6.8)	51 (20.3)	183 (72.9)	medium			
Inadequate	17 (26.6)	10 (15.6)	37 (57.8)				
**BRIEF**							
Adequate					37 (16.8)	183 (83.2)	medium
Marginal					10 (16.4)	51 (83.6)	
Inadequate					17 (50.0)	17 (50.0)	

* Effect size measurements: for BRIEF by age, eta squared; CHLT-6 by age, Cohen's d; BRIEF by gender, education, language, self-reported confidence and CHLT-6, Cramérs V; CHLT-6 by gender, education, language and self-reported confidence, Phi coefficient. Interpretations as per Cohen 1998 [25]

**Percentages are row percentages

Relationships between patient characteristics and the measures of health literacy were explored and results are shown in Table 3. Of note, the relationship between participants’ own assessment of their reading and writing ability and their self-assessment of health literacy in the BRIEF showed a large effect, with a considerable proportion of respondents who had reservations in their abilities scoring ‘inadequate’ on the BRIEF (47% of those with reservations with reading and 42% of those with reservations in writing).

*Reference for footnote for table 3: [25]

### Free text analysis

There were 166 notes recorded in the free text question of the survey by volunteers regarding barriers or issues with completion of the screening tool, and any other feedback offered by participants.

Comments regarding patient confidence were often linked with successful survey completion and willingness to participate. In some instances, however, patients would not have been able to complete the survey without assistance. Volunteers noted 22 instances of family assistance, with this predominantly taking the form of interpreting for patients whose first language was not English: *“[Patient’s] command of English was insufficient to ask most questions”*.

The most common barrier noted by volunteers related to timing – with 19 recorded instances where patients were called into their clinic appointment, treatment or tests mid-way through completing the survey. Low English language ability, as noted above, was also a significant barrier to survey completion. In some instances where family assistance was not available, patients were unable to complete the survey, or specific survey items *“Decided not to answer questions 2 and 3 of the BRIEF literacy screening tool - some difficulties with English/language barrier, so did not feel comfortable answering these”.* Often difficulties were specific to a particular instrument or item: “*CHLT-6 note: patient not able to understand/comprehend questions”*. There were 18 specific issues recorded relating to completion of the CHLT-6 and 10 relating to the BRIEF.

Volunteers noted other information, which they felt impacted patients’ ability to complete the health literacy screening tools, such as: recent diagnosis, first day of treatment, first time attending the hospital, or feeling overwhelmed: *“Patient recently diagnosed so hasn't had a great deal of experience with medical terms”.*

Patients also provided feedback, particularly on the topic of health literacy, including requests for more information and preferences for format of information (verbal vs written). Patients felt the health service should be responsible for ensuring they understood every element of what was happening to them. In particular, it was mentioned that shock or other issues relating to treatment impacted their ability to absorb information, the use of ‘medical jargon’ was a problem, and they struggled with navigating the health service and finding reliable medical information online: “*Patient said she had difficulty accepting some information due to shock value and it had to be provided in stages at a level she could comprehend at the time”.*

## DISCUSSION AND CONCLUSION

This study aimed to identify the characteristics and proportion of outpatients with low health and cancer literacy visiting a specialist cancer hospital using a general health literacy instrument and a cancer-specific health literacy instrument. Overall, the proportion of patients identified as having low health literacy by each tool was somewhat low, although more patients were identified as having inadequate health literacy by the CHLT-6 (n=66, 21%), as compared with the BRIEF (n=43, 13%). At first these findings seem reasonable, as the BRIEF asks respondents to discuss their ability to read, understand, learn and apply medical information, whereas the CHLT-6 is designed as a knowledge/recall questionnaire of key cancer terms, which may be more difficult in ‘real world’ situations [1, 15]. However, review of the data identified a proportion of patients who scored ‘adequate’ on the BRIEF but concurrently scored ‘inadequate’ on the CHLT-6 (*n*=37, 16.8). These individuals report no difficulty managing their care according to the BRIEF, but in practice have difficulty with simple cancer terminology as used in the CHLT-6. This indicates that what was measured by the CHLT-6 as cancer health literacy may differ from what was measured as general health literacy by the BRIEF, however it is unclear how or why this is so. Further assessment is needed, to clarify what was measured, and whether patient self-report responses were impacted by factors such as perception and/or lack of understanding. More importantly, differences in those identified as having inadequate health literacy make it difficult to determine which system-level changes or universal precaution approaches would be the most useful and appropriate, given that results from the CHLT-6 indicate that approximately one-fifth of cancer patients may have inadequate understanding of their disease.

Other studies have found similar issues when comparing different measures of health literacy, namely, that tools differ in who they classify as having inadequate health literacy [17, 26]. A recent assessment of self-administered health literacy tools concluded that no current ‘gold standard’ exists, meaning there is no ‘true’ reference with which to establish comparison [17, 27]. Further, experts recommend that the construct of health literacy be extensively reviewed before adequate and reliable tools can be developed [27]. This is perhaps unsurprising, given the wide variety of definitions of health literacy, and skills and behaviours described within conceptual models of health literacy [2, 4, 17].

The corollary of differing predictive values of screening tools tested is that they have limited clinical utility, unless they can be clearly mapped to a cohesive underlying construct of health literacy [17]. Our results reflected previous research which demonstrated that identification of low or inadequate health literacy depends heavily on the tool selected [26]. Previous studies have further suggested that health literacy is best assessed using a battery of measures [17]. However, in our study, even using short screening tools, a large proportion of people attending clinics could not be approached due to the volunteer interview time capacity, and pragmatic limitations such as patient volume and movement. Despite screening being undertaken verbally to facilitate participation by those who lack reading and writing skills, a proportion of people still declined to participate. Low literacy is known to be associated with shame and stigma, and individuals may avoid situations where their lack of skills may be detected [28]. Using verbal screening may not overcome this issue. A lack of strong English language skills also made it difficult for patients to complete verbal surveys, even when family provided interpreting assistance. Using longer surveys, or a battery of different tools to overcome current health literacy construct issues, would likely amplify these barriers.

Patient comments highlighted the obstacles they experienced in regards to understanding and effectively managing personal health including: being overwhelmed with information and the difficulty of understanding medical terminology when newly diagnosed. Patients have previously described similar personal, social, environmental, and health service barriers impacting on their ability to manage their health [4]. Research outside of the health literacy field has likewise identified factors which significantly influence an individual’s capacity to engage with health services, such as: overly complex healthcare system design, busy or loud environments, or receiving information when unwell or in shock [13, 29, 30]. Despite this, many conceptual models of health literacy do not adequately account for these factors, instead, focus is placed on an individual’s characteristics and abilities, predominantly literacy and numeracy, thereby promoting a ‘individual deficiency paradigm’ of health literacy that ignores or diminishes external contributing factors [16]. Concernedly, these models underpin most health literacy measurement tools currently in use [16]. In our study participants were comfortable identifying reservations with reading and writing, and did not consider these obstacles or barriers to managing their health.

Newer models of health literacy now include the environmental, situational and social demands and complexities faced by patients outside of health information and communication [16]. Use of these models which comprehensively capture all elements facing patients within the healthcare setting would be more useful in developing screening tools, and ostensibly avoid issues identified in our study, where the same patients were categorised as having inadequate or adequate health literacy depending on the measure used.

Further, 32% and 35% of people in our study who reported having reservations with reading and writing, respectively, were categorised through self-report as having adequate health literacy by the BRIEF. Importantly, this demonstrates that people who have difficulty with reading and writing literacy skills, still feel competent to understand and manage their health. Many universal precaution approaches within the health service setting focus predominantly on improving written resources, or health professional communication. However these approaches are only useful if people feel comfortable or proficient in their reading and writing skills, or if a lack of written information, or doctor-patient communication is a problem [16]. A broader range of elements should therefore be considered when developing system-level responses to health literacy, such as environmental factors (ambient noise levels; privacy; time-pressures), situational factors (timing; volume of information) social factors (cultural context; emotional context; presence of family or friends). Additionally, a wider range of interventions and information delivery mediums should be considered than written materials, or physician communication skills training.

Our study identified firsthand how current inconsistencies within the field of health literacy limits its effective application in practice. Introducing newer models of health literacy which encompass social, environmental and situational factors and phasing out older models and screening tools is recommended. Further, system-level and universal precaution approaches should focus on more than improving health information and communication.

### Limitations

While we administered the surveys verbally to reduce any literacy or language barriers, the validity of the BRIEF and CHLS in populations with low or limited English has not been established. Some of the terms included in the CHLT-6 such as ‘inoperable’ may be discussed in more lay language by healthcare practitioners; hence patients may not be aware of them. Cross-sectional survey data were collected from a single site, limiting generalisability. While the response rate was only 14%, unpublished data from the study site indicates the sample was largely representative of the population in regards to age, gender and disease type.

### Conclusion

Undertaking a cross-sectional survey to identify low health literacy in this context was not clinically useful, predominantly because the different screening measures used did not identify the same population as having low or inadequate health literacy to allow for appropriate clinical response. Importantly, these findings support calls for the definition of health literacy to be reviewed and updated to ensure greater consistency and accuracy. Further, as the construct of health literacy is complex, multi-faceted, and must also take into account a variety of environmental, social and situational factors, perhaps health service performance assessment and intervention, rather than individual patient screening may be more achievable and feasible for improving health literacy of patient populations.

#### Practice implications

Current screening tools appear to identify differing constructs of health literacy, which may impact the development of an appropriate health system response. The concept of health literacy requires more clarity in order to better understand measurement outcomes.

## Supplementary information

ESM 1 (DOCX 12.7 kb)

ESM 1 (DOCX 14.1 kb)

## Data Availability

Available upon request.

## References

[1] Chew LD, Bradley KA, Boyko EJ (2004). Brief questions to identify patients with inadequate health literacy. Health.

[2] Freedman DA, Bess KD, Tucker HA, Boyd DL, Tuchman AM, Wallston KA (2009) Public health literacy defined. Am J Prev Med 36 10.1016/j.amepre.2009.02.001 19362698

[3] Heinrich C (2012). Health literacy: The sixth vital sign. J Am Acad Nurse Pract.

[4] Jordan JE, Buchbinder R, Osborne RH (2010). Conceptualising health literacy from the patient perspective. Patient Educ Couns.

[5] Nutbeam D (2008) The evolving concept of health literacy. Soc Sci Med 67 10.1016/j.socscimed.2008.09.050 18952344

[6] Speros C (2005) Health literacy: concept analysis. J Adv Nurs 50 10.1111/j.1365-2648.2005.03448.x 15926968

[7] Berkman ND, Sheridan SL, Donahue KE, Halpern DJ, Crotty K (2011). Low health literacy and health outcomes: an updated systematic review. Ann Intern Med.

[8] DeWalt DA, Berkman ND, Sheridan S, Lohr KN, Pignone MP (2004). Literacy and health outcomes. J Gen Intern Med.

[9] Herndon JB, Chaney M, Carden D (2011). Health literacy and emergency department outcomes: a systematic review. Ann Emerg Med.

[10] Hill SJ, Sofra TA (2018). How could health information be improved? Recommended actions from the victorian consultation on health literacy. Aust Health Rev.

[11] Australian Institute of Health and Welfare (2019) Cancer in Australia 2019. Cancer series no.119. Cat. no. CAN 123, Canberra, AIHW

[12] Dumenci L, Matsuyama RK, Riddle DL, Cartwright L, Siminoff LA (2018). Validation of the cancer health literacy test-30 for populations without cancer. HLRP: Health Lit Res and Pract.

[13] Organisation for Economic Co-operation and Development (2015) Reviews of health care quality: Australia 2015

[14] Davis TC, Williams MV, Marin E, Parker RM, Glass J (2002). Health literacy and cancer communication. CA: Cancer J Clin.

[15] Dumenci L, Matsuyama R, Riddle DL, Cartwright LA, Perera RA, Chung H (2014). Measurement of cancer health literacy and identification of patients with limited cancer health literacy. J Health Commun.

[16] Okan O, Bauer U (2019) International handbook of health literacy: research, practice and policy across the life-span. Policy Press

[17] Haun J, Luther S, Dodd V, Donaldson P (2012). Measurement variation across health literacy assessments: implications for assessment selection in research and practice. J Health Commun.

[18] Haun JN, Valerio MA, McCormack LA, Sørensen K, Paasche-Orlow MK (2014). health literacy measurement: an inventory and descriptive summary of 51 instruments. J Health Commun.

[19] Lorini C, Lastrucci V, Paolini D, Bonaccorsi G (2020). Measuring health literacy combining performance-based and self-assessed measures: the roles of age, educational level and financial resources in predicting health literacy skills. A cross-sectional study conducted in Florence (Italy). BMJ Open.

[20] Haun J, Noland-Dodd V, Varnes J, Graham-Pole J, Rienzo B, Donaldson P (2009). Testing the BRIEF health literacy screening tool. Fed Prac.

[21] Harris PA, Taylor R, Thielke R, Payne J, Gonzalez N, Conde JG (2009). Research electronic data capture (REDCap)—A metadata-driven methodology and workflow process for providing translational research informatics support. J Bio Inform.

[22] R Development Core team (n.d.) R: a language and environment for statistical computing, Vienna ARFfSCAfhwR-po

[23] Forman J, Damschroder L (2007) Qualitative content analysis. In: Empirical methods for bioethics: a primer. Emerald Group Publishing Limited, pp 39–62

[24] Microsoft Office Professional Plus (2010) MECS

[25] Cohen J (1992). A power primer. Psychol Bull.

[26] Barber MN, Staples M, Osborne RH, Clerehan R, Elder C, Buchbinder R (2009) Up to a quarter of the Australian population may have suboptimal health literacy depending upon the measurement tool: results from a population-based survey. Health Promot Int 24 10.1093/heapro/dap022 19531559

[27] O'Neill B, Daniela G, Ignacio R-C, Sue Z, Jose V (2014). An overview of self-administered health literacy instruments. PLOS One.

[28] Parikh NS, Parker RM, Nurss JR, Baker DW, Williams MV (1996). Shame and health literacy: the unspoken connection. Patient Educ Couns.

[29] Kessels RP (2003). Patients’ memory for medical information. J Royal Soc Med.

[30] Williams MV, Davis T, Parker RM, Weiss BD (2002). The role of health literacy in patient-physician communication. Fam Med.

