# Supplementary material for: Identification of low health and cancer literacy in oncology patients: a cross-sectional survey
Source: Support Care Cancer. 2021 May 3;29(11):6605–12. doi: 10.1007/s00520-021-06164-2 (PMC8464552; doi:10.1007/s00520-021-06164-2)
Supplement: Supplementary file 1 — (DOCX 12.7 kb) [file 520_2021_6164_MOESM1_ESM.docx]

**Table 3:** BRIEF Health Literacy questionnaire items (n=345)

|  | **Always** | **Often** | **Sometimes** | **Occasionally** | **Never** | **Missing** |
| --- | --- | --- | --- | --- | --- | --- |
| How often do you have someone help you read hospital materials? | 29 (8.4) | 18 (5.2) | 24 (7.0) | 36 (10.4) | 237 (68.7) | 1 (0.3) |
| How often do you have problems learning about your medical condition because of difficulty understanding written information? | 7 (2.0) | 15 (4.3) | 46 (13.3) | 47 (13.6) | 220 (63.8) | 10 (2.9) |
| How often do you have a problem understanding what is told to you about your medical condition? | 10 (2.9) | 11 (3.2) | 44 (12.8) | 75 (21.7) | 196 (56.8) | 9 (2.6) |
|  | **Not at all** | **A little bit** | **Somewhat** | **Quite a bit** | **Extremely** | **Missing** |
| How confident are you at filling out medical forms by yourself? | 27 (7.8) | 22 (6.4) | 26 (7.5) | 57 (16.5) | 206 (59.7) | 7 (2.0) |
