# Supplementary material for: Identification of low health and cancer literacy in oncology patients: a cross-sectional survey
Source: Support Care Cancer. 2021 May 3;29(11):6605–12. doi: 10.1007/s00520-021-06164-2 (PMC8464552; doi:10.1007/s00520-021-06164-2)
Supplement: Supplementary file 2 — (DOCX 14.1 kb) [file 520_2021_6164_MOESM2_ESM.docx]

|  |  |  |  |
| --- | --- | --- | --- |
| **The normal range for haemoglobin for a male is 13.3-17.2 g/dl. Joe's haemoglobin is 9.7 g/dl. Is Joe within the normal range?** | | |  |
| Yes | 41 | 11.9 | 12.5 |
| *No | 275 | 79.7 | 83.6 |
| Unsure | 13 | 3.8 | 4.0 |
| Missing | 16 | 4.6 |  |
| **A biopsy of a tumour is done to…** | |  |  |
| Remove it | 13 | 3.8 | 4.0 |
| *Diagnose it | 296 | 85.8 | 90.5 |
| Treat it | 12 | 3.5 | 3.7 |
| Unsure | 6 | 1.7 | 1.8 |
| Missing | 8 | 2.3 |  |
| **If a patient has stage 1 cancer, it means the cancer is…** | | |  |
| *Localized | 277 | 80.3 | 85.0 |
| In nearby organs | 16 | 4.6 | 4.9 |
| In distant sites | 9 | 2.6 | 2.8 |
| Unsure | 24 | 7.0 | 7.4 |
| Missing | 19 | 5.5 |  |
| **The role of a physiotherapist is to talk to a patient about emotional needs.** | | |  |
| True | 34 | 9.9 | 10.5 |
| *False | 286 | 82.9 | 88.5 |
| Unsure | 3 | 0.9 | 0.9 |
| Missing | 22 | 6.4 |  |
| **A tumour is considered 'inoperable' when it cannot be treated with…** | | |  |
| Radiation therapy | 10 | 2.9 | 3.1 |
| *Surgery | 253 | 73.3 | 78.8 |
| Chemotherapy | 32 | 9.3 | 10.0 |
| Unsure | 26 | 7.5 | 8.1 |
| Missing | 24 | 7.0 |  |
| **Sally will get radiation therapy once a day, Monday through Friday. If Sally has therapy for 4 weeks, how many times will she get radiation therapy?** | | |  |
| 5 | 4 | 1.2 | 1.3 |
| 15 | 5 | 1.4 | 1.6 |
| *20 | 306 | 88.7 | 95.9 |
| Unsure | 4 | 1.2 | 1.3 |
| Missing | 26 | 7.5 |  |
| * indicates correct answer |  |  |  |

**Table 4**: CHLT-6 Cancer Health Literacy Items (n=345)
